# Detection of *Ascaris lumbricoides* infection by ABA-1 coproantigen ELISA

**DOI:** 10.1371/journal.pntd.0008807

**Published:** 2020-10-15

**Authors:** Ole Lagatie, Ann Verheyen, Kim Van Hoof, Dax Lauwers, Maurice R. Odiere, Johnny Vlaminck, Bruno Levecke, Lieven J. Stuyver

**Affiliations:** 1 Janssen Global Public Health, Janssen R&D, Beerse, Belgium; 2 Janssen Biologics, Janssen R&D, Beerse, Belgium; 3 Kenya Medical Research Institute, Centre for Global Health Research, Kisumu, Kenya; 4 Department of Virology, Parasitology and Immunology, University of Ghent, Merelbeke, Belgium; McGill University, CANADA

## Abstract

Intestinal worms, or soil-transmitted helminths (STHs), affect hundreds of millions of people in all tropical and subtropical regions of the world. The most prevalent STH is *Ascaris lumbricoides*. Through large-scale deworming programs, World Health Organization aims to reduce morbidity, caused by moderate-to-heavy intensity infections, below 2%. In order to monitor these control programs, stool samples are examined microscopically for the presence of worm eggs. This procedure requires well-trained personnel and is known to show variability between different operators interpreting the slides. We have investigated whether ABA-1, one of the excretory-secretory products of *A*. *lumbricoides* can be used as a coproantigen marker for infection with this parasite. Polyclonal antibodies were generated and a coproantigen ELISA was developed. Using this ELISA, it was found that ABA-1 in stool detected *Ascaris* infection with a sensitivity of 91.5% and a specificity of 95.3%. Our results also demonstrate that there is a correlation between ABA-1 levels in stool and *A*. *lumbricoides* DNA detected in stool. Using a threshold of 18.2 ng/g stool the ABA-1 ELISA correctly assigned 68.4% of infected individuals to the moderate-to-heavy intensity infection group, with a specificity of 97.1%. Furthermore, the levels of ABA-1 in stool were shown to rapidly and strongly decrease upon administration of a standard anthelminthic treatment (single oral dose of 400 mg albendazole). In an *Ascaris suum* infection model in pigs, it was found that ABA-1 remained undetectable until day 28 and was detected at day 42 or 56, concurrent with the appearance of worm eggs in the stool. This report demonstrates that ABA-1 can be considered an *Ascaris* -specific coproantigen marker that can be used to monitor infection intensity. It also opens the path for development of point-of-care immunoassay-based tests to determine *A*. *lumbricoides* infection in stool at the sample collection site.

## Author summary

Intestinal worms are one of the most common infections in tropical and subtropical parts of the world. The roundworm *Ascaris lumbricoides* is the most prevalent and efforts are ongoing to use preventive chemotherapy to reduce both prevalence and intensity of this infection. To monitor these programs, stool-based microscopy is currently used. We have investigated the possibility of using ABA-1, an abundantly secreted protein from the worm, as a biomarker in stool of infected individuals. We have developed an ELISA and using this assay determined that ABA-1 as stool biomarker had a sensitivity of 91.5% and a specificity of 95.3% to detect infection with *A*. *lumbricoides*. We also showed that ABA-1 in stool rapidly and strongly decreased upon administration of a standard anthelminthic treatment. The main asset of this novel stool biomarker is its potential to be used in point-of-care immunoassay-based tests to determine *A*. *lumbricoides* infection in stool at the sample collection site.

## Introduction

According to the World Health Organization (WHO), approximately 1.5 billion people, or 24% of the world’s population, are infected with soil-transmitted helminths (STH) worldwide [1]. This group of parasites comprises the intestinal worms *Ascaris lumbricoides*, *Trichuris trichiura*, and the hookworm species *Ancylostoma duodenale* and *Necator americanus*. They pose a major threat to public health in large parts of the world. Children and women of childbearing age are at highest risk of developing morbidity, which is mainly associated with moderate-to-heavy intensity (M&HI) infections [2]. It is therefore the goal of the WHO to reduce the prevalence of preschool and school-aged children with STH infections of M&HI below 2% by 2030 [3]. To reach this goal, so-called preventive chemotherapy (PC) programs have been implemented in which anthelminthic drugs (e.g. mebendazole or albendazole) are administered to school-aged children, regardless of their infection status [4]. Global control efforts for soil-transmitted helminthiasis are an essential part of the Sustainable Development Goals (SDGs) put forward by the WHO where it contributes to achieve goal # 3: good health and well-being.

Monitoring of the current programs and decision-taking is currently based on the detection and quantification of STH eggs in a stool smear using a compound microscope, the so called Kato-Katz thick smear technique [5–8]. This procedure however lacks standardization, is time-consuming and requires trained personnel and specific laboratory infrastructure. New diagnostic tools are therefore highly desired. Target Product Profiles (TPPs) describing the specific requirements for such new diagnostic approaches required for different use-cases were published and the identification of so-called coproantigens (i.e. antigen biomarkers present in stool) will enable the development of novel diagnostic tools to assess progress against program goals (use-case 2) [5].

In the veterinary world, the use of coproantigen detection to diagnose helminth infection has become widely adopted [9–11]. Diagnosis of human helminth infections based on coproantigen detection has also been investigated for e.g. *Fasciola* species, *Strongyloides stercoralis* and *Taenia solium* [12–14]. To our knowledge, assays to detect roundworm coproantigens have been limited to *Toxocara* species and no specific proteins have been identified that are used as coproantigen for *Ascaris* species, such as *A*. *suum* in pigs and *A*. *lumbricoides* in humans [11, 15].

A few studies have explored the excretory-secretory products from different stages of *A*. *suum* [16–18]. These analyses were however based on isolated worms or larvae and to our knowledge no reports have described whether the identified proteins were readily detectable in feces of individuals with active infections. We have therefore investigated whether ABA-1, a protein that is known to be released by adult *Ascaris* worms and some larval stages, could be detected in fecal samples using a coproantigen ELISA [19]. ABA-1 is a well-known allergen of approximately 14 kDa and is among the most abundant proteins synthesized by the nematode parasite *Ascaris* [19–22].

## Materials and methods

### Ethics approval and consent to participate

Human samples from Kenya were collected as part of a field study in Kenya. The study was approved by the KEMRI Scientific and Ethics Review Unit (SERU), Nairobi, Kenya (Protocol # KEMRI/SERU/CGHR/102/3554). Since all study participants were minors, informed consent forms were signed by parents/guardians of the study participants, and verbal assents were obtained from all study participants.

All animal experiments were conducted in accordance with the E.U. Animal Welfare Directives and VICH Guidelines for Good Clinical Practice. Ethical approval to conduct the studies was obtained from the Ethical Committee of the Faculty of Veterinary Medicine, Ghent University.

### Human study samples

Plasma and urine samples from Kenya were collected as part of a field study. The study was approved by the KEMRI Scientific and Ethics Review Unit (SERU), Nairobi, Kenya (Protocol # KEMRI/SERU/CGHR/102/3554). Since all study participants were minors, signed informed consent forms were obtained from their parents/guardians, and verbal assents were obtained from all study participants. This study was undertaken in the former Nyanza province, in the southwest part of Kenya, with collections in the Kisumu county (high *S*. *mansoni* prevalence area) and Siaya county (high STH prevalence area). Stool samples were collected in order to determine the STH and *Schistosoma mansoni* infection status, based on qPCR-based quantification of helminth DNA present in stool. A total of 474 participants that donated stool samples were included in this study. Of these participants, 71 were found to be positive for *A*. *lumbricoides*, 30 for *T*. *trichiura*, 16 for hookworm, and 82 for *S*. *mansoni*, based on qPCR detection of helminth DNA in stool (see below). After the cross-sectional collection of samples, a total of 22 *A*. *lumbricoides* infected individuals were treated with a single dose of albendazole (400mg) and stool samples were collected at day 6, day 12 and day 24 post-treatment. Infection status at each timepoint was determined by qPCR-based quantification of helminth DNA present in stool.

### Pig study samples

Twenty-five pigs were selected for this study. A total of 5 pigs served as uninfected controls. A first group of 10 pigs received a trickle infection of 20 infective *A*. *suum* eggs for a total of 3 times per week. A second group of 10 pigs received a dose of 100 infective *A*. *suum* eggs three times per week. Doses were administered orally in a food bolus to each pig individually in order to mimic a low natural exposure. Pigs received infection doses for a total of 6 weeks. In total this corresponded with 360 or 1800 infective eggs given to each pig over the course of the trial depending on their respective infection group. On day 0, 14, 28, 42 and 56, stool was collected. These samples were used to determine the number of *A*. *suum* eggs per gram of feces using the Mini-FLOTAC method and to assess ABA-1 coproantigen levels [23].

### Stool-based assessment of helminth infection

Helminth infection in humans was assessed on stool samples using the Kato-Katz procedure and/or qPCR analysis, as described before [24, 25]. Briefly, stool samples stored in ethanol were first subject to centrifugation at 9,000 g for 1.5 minutes and the resulting pellet was used for DNA extraction using the DNeasy PowerSoil Kit (Qiagen, Germany) according to the manufacturer’s instructions. *A*. *lumbricoides* DNA was quantified by qPCR using primers and probe targeting the *A*. *lumbricoides* ITS1. In case of a discordant infection status (positive *vs*. negative) between egg count and qPCR analysis, qPCR was repeated on a second stool aliquot from the same individual and qPCR results were confirmed. It was therefore decided to use the qPCR data as the reference data set.

For determination of a qPCR-based cut-off for moderate infection, linear regression analysis was performed on log-transformed qPCR data (in cps/rxn) and Kato-Katz data (in epg). Based on this analysis, a qPCR result of 700 cps/rxn was found to correspond to 5,000 epg, i.e. the boundary for moderate infection intensity for *A*. *lumbricoides* (S1 Fig).

### Preparation of stool samples for coproantigen ELISA

Stool sample aliqouts—frozen at -80C - were allowed to thaw at room temperature and an aliquot of approx. 250 mg was transferred to a Powerbead tube (Qiagen, Germany). Samples were further processed by addition of 1 mL of ice-cold PBS followed by bead beating in a tissue homogenizer (Bertin, France) for 1 minute at 6,500 rpm. Stool extracts were centrifuged at 23,000 x g for 15 minutes at 4°C and supernatant was transferred to a protein LoBind tube (Eppendorf, Germany) and stored at -20°C till further use. Where indicated the bead beating step was omitted from the procedure.

### Preparation of pseudocoelomic fluid of *A*. *suum* and a protein extract of adult *A*. *suum* worms

Adult *A*. *suum* worms were collected from naturally infected pigs at the local slaughterhouse. Collected adult worms were washed three times in tap water and the pseudocoelomic fluid was collected after cutting of the posterior tip off the worm. The pseudocoelomic fluid was then centrifuged for 15 min at 10,000 × *g* and 4°C and the supernatant filtered (0.22 μm) and stored at -80°C until use. Adult worm protein extracts were produced as described before [26]. Briefly, extracts were prepared by grinding the adult worms with a mortar and pestle that was placed in a bath of liquid nitrogen. The worm powder was transferred to a 15 ml tube and mixed with PBS and proteinase inhibitor cocktail (1:100) (Sigma, Diegem, Belgium). The homogenate was then thoroughly mixed by inversion at 4°C for two hours followed by centrifugation for 30 min at 10,000 x g at 4°C. The supernatant (PBS extract) was removed and sterilized by filtration (0.22μm) and stored at -80°C until use. Protein concentration was determined by the BCA method (Pierce, Rockford, USA).

### Production of recombinant ABA-1

An expression vector with the ABA-1 sequence (GenBank ID: AAD13651.1) was constructed and used to produce recombinant his-tagged ABA-1 in a bacterial expression system (Eurogentec, Belgium). The produced recombinant protein was purified using chromatography His-tag based column under denaturing conditions. After elution, to remove urea and imidazole, the purified fraction was dialyzed at 4°C against a refolding buffer containing decreasing concentrations of urea and finally against PBS buffer. Approximately 13 mg of the purified His-tagged protein was obtained and this purified protein was found by SDS-page to have a molecular weight of approximately 32 kDa, similar to the theoretical Mw (S2 Fig). A total volume of 11.9 mL at a concentration of 1.16 mg/mL was stored at -20°C. This stock solution was further diluted to 10 μg/mL in PBS, followed by a 1:2 dilution with glycerol to a final concentration of 5 μg/mL. Aliquots of this working solution were stored at -20°C till further use.

### Generation of anti-ABA-1 polyclonal antibody

For the preparation of the polyclonal antibody, rabbits were immunized using 4 different injections with 100 μg of the recombinant ABA-1 with a non-Freund adjuvant, based on the Speedy 28-day program (Eurogentec, Belgium). Antibodies were purified from 70 mL serum of the animal with the highest titer using an antigen-specific affinity purification. A total of 24.3 mg of affinity purified IgG was dissolved in 6.2 mL of PBS with 0.01% thimerosal and 0.1% BSA. Aliquots of the purified antibodies, 1:2 diluted with glycerol were stored at -20°C.

HRP conjugated polyclonal antibody was produced (Eurogentec, Belgium) by covalently conjugating Horse Radish Peroxidase (Sigma, USA) using NaIO_4_. A total of 5 mg of antibody coupled to HRP was obtained in a volume of 2 mL PBS. Aliquots of the HRP conjugated antibodies, 1:2 diluted with glycerol were stored at -20°C.

### Preparation of ABA-1 calibration curve

A stock solution of the recombinant ABA-1 was prepared at a concentration of 5 μg/mL in PBS. Calibration samples were generated by first diluting this stock solution in block buffer (Superblock + 0.05% Tween) to a concentration of 100 ng/mL and then 1/3 serial dilutions were generated in block buffer till a concentration of 0.137 ng/mL. Also, a blank sample containing only block buffer was included.

### Enzyme linked immunosorbent assay

The ELISA for coproantigen detection was performed as follows. Flat bottomed polystyrene plates (Maxisorp Immuno Plate, Nunc, Denmark) were coated with purified rabbit anti-ABA-1 IgG diluted to 0.8 μg/mL in PBS and incubated for 2 hours at room temperature. Next, after washing with PBS-T (PBS with 0.05% Tween-20) the plate was blocked with block buffer (Superblock + 0.05% Tween) for 1 hour at room temperature. Calibration samples or stool extracts, diluted 5-fold in block buffer, were added and incubated for 1 hour at room temperature. Subsequently, plates were washed 3 times with PBS-T and purified rabbit anti-ABA-1 IgG conjugated with HRP was added at a concentration of 2.5 μg/mL and incubated for 1 hour at room temperature. Plates were washed 5 times with PBS-T and color development was done using Sureblue TMB (KPL, the Netherlands). The reaction was stopped after 10 minutes using 1N HCl and optical density was measured at 450 nm.

Calibration curves were analyzed using 5-parameter logistic regression in SoftMax Pro version 7.1 and concentrations in the sample extracts were determined by back-calculating the values obtained in the ELISA using this calibration curve and correction for the dilution. Concentrations of ABA-1 in stool were expressed as ng ABA-1 / g stool by multiplying the concentration in the 5-fold diluted extract (in ng/mL) by 20 (based on an estimated 250 mg in 1 mL of extraction buffer).

### Statistical analysis

Calibration curves for ABA-1 were generated using 5-Parameter Logistic regression and ABA-1 concentration in unknown and calibration samples were back-calculated using this calibration curve. Accuracy and precision were determined by analyzing 4 calibration curves on 3 consecutive days (also called runs). Accuracy at each calibration point was determined by dividing the average back-calculated value by the nominal value. For each calibration point, the within-run coefficient of variability (CV) was calculated by dividing the average standard deviation (sd) of the three days by the average concentration for that point. The between-run CV was calculated as the sd of the average of each day divided by the average concentration for that point. For evaluation of the correlation between ABA-1 coproantigen ELISA and qPCR-based detection of *A*. *lumbricoides* DNA in stool, linear regression analysis was performed on log-transformed data. For determination of the association between ABA-1 positive stool and a specific infection, contingency tables were prepared, Fisher’s exact test was performed, and Odds ratio was calculated. For association with *A*. *lumbricoides*, all data were used, for association with any other infection, only data from *A*. *lumbricoides* negative subjects were used. All analyses were performed using GraphPad Prism version 7.00.

## Results

**Fig 1:**
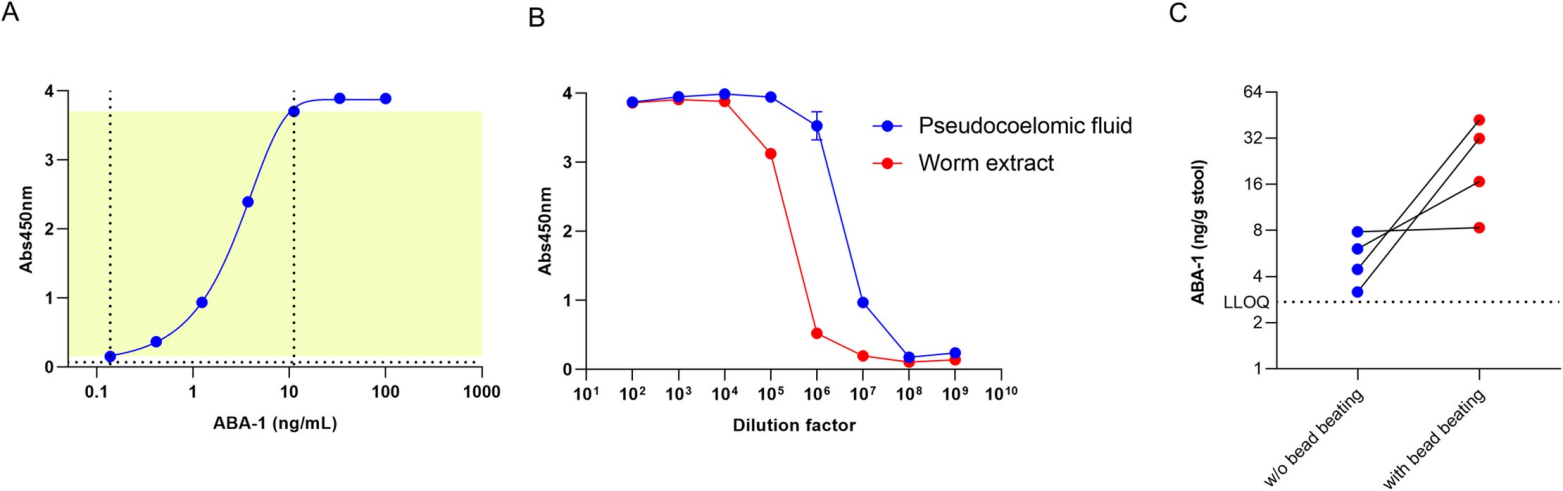
**A. Calibration curve for the ABA-1 coproantigen ELISA.** Yellow area indicates the dynamic range of the assay, left vertical dotted line corresponds to the LLOQ (0.137 ng/mL), right vertical dotted line to the ULOQ (11.1 ng/mL), the horizontal dotted line corresponds to the background signal detected in blanks. **B.** Analysis of a serial dilution of pseudocoelomic fluid (blue circles) and an adult worms extract (red circles) on the ABA-1 ELISA. **C.** ABA-1 coproantigen ELISA using stool extracts with and without bead beating indicates that a cell destruction step is needed to release ABA-1 in the stool supernatant.

An ELISA was developed using affinity-purified anti-ABA-1 polyclonal rabbit antiserum (Fig 1A). Performance characteristics of the ELISA are presented in S1 Table. The lower limit of quantification (LLOQ) of the assay was defined at 0.137 ng/mL, the upper limit of quantification (ULOQ) was found to be 11.1 ng/mL. Since stool extracts were prepared by resuspending approximately 250 mg stool in 1 mL extraction buffer, and extracts were diluted 5-fold before being analyzed, this corresponded to an LLOQ of 2.74 ng/g stool and an ULOQ of 222 ng/g stool. In order to demonstrate that the ELISA was able to detect native ABA-1 produced *in vivo* by the worm, pseudocoelomic fluid of *A*. *suum* and a protein extract of adult *A*. *suum* worms were tested in the ELISA (Fig 1B). Both samples were found to have very high levels of ABA-1, with the worm extract about 1 mg/mL and the pseudocoelomic fluid about 10 mg/mL.

To assess whether ABA-1 could be detected in stool and whether this was associated with eggs or excreted by the worm, a subset of four *A*. *lumbricoides* qPCR positive stool samples were tested. An aliquot of each sample was processed either with or without a bead beating step in the procedure (Fig 1C). This analysis revealed that bead beating results in a strong increase in the amount of ABA-1 protein detected in the stool supernatant by the coproantigen ELISA.

**Fig 2:**
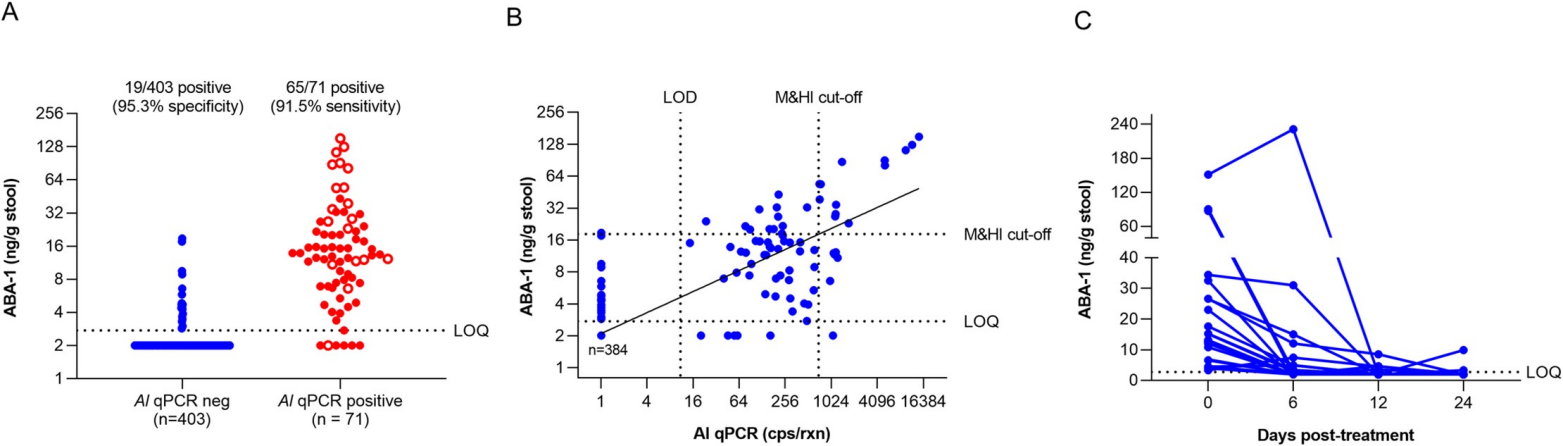
**A.** Assessment of ABA-1 coproantigen levels in stool samples from a cohort of 474 subjects collected in Kenya, stratified according to their *A*. *lumbricoides* qPCR result. Open circles indicate subjects with M&HI infection. **B.** Correlation between ABA-1 coproantigen levels and *A*. *lumbricoides* DNA detection in stool collected in Kenya (expressed in *A*. *lumbricoides* copies/reaction). Based on the linear regression, a cut-off of 18.4 ng/g stool was defined to identify subjects with M&HI infection. Moderate infection was defined as >700 cps/rxn (see Materials and Methods). **C.** Effect of treatment with albendazole on the presence of ABA-1 in stool. Stool samples were collected before (Day 0) and at different timepoints (6, 12 and 24 days) after treatment with albendazole.

Using this ELISA, including the bead beating procedure, the concentration of ABA-1 was determined in a stool sample set of 474 individuals from the Kisumu region in Kenya, an area that is endemic for *A*. *lumbricoides*. Of the 71 individuals that were qPCR positive for *A*. *lumbricoides*, 65 had ABA-1 levels in stool supernatant above LLOQ, corresponding to a sensitivity of 91.5% (Fig 2A). Of the 403 *A*. *lumbricoides* qPCR negative individuals, 19 were found positive in the ABA-1 coproantigen ELISA, corresponding to a specificity of 95.3%. Although 18 subjects of the *A*. *lumbricoides* negative group were found to be positive for *T*. *trichiura*, 9 for hookworm and 75 for *S*. *mansoni*, there was no association with ABA-1 detection in stool and either hookworm or *S*. *mansoni* infection (P > 0.05), while a weak association was found with *T*. *trichiura* infection (P = 0.046). To assess whether possible cross-reaction with a *T*. *trichiura* protein was causing this, blast analysis was performed on the *T*. *trichiura* proteome, but no homologs could be found (S1 Data). The association with *A*. *lumbricoides* infection, however, was highly significant (P < 0.0001). Odds ratio for a positive ABA-1 result was 219 (95% CI: 84.7–540) for *A*. *lumbricoides* infection, while this was 4.61 (95% CI: 1.30–16.4) for *T*. *trichiura*, 2.61 (95% CI: 0.224–16.2) for hookworm and 0.501 (95% CI: 0.113–2.07) for *S*. *mansoni*. Furthermore, there was a highly significant positive correlation between *A*. *lumbricoides* DNA copies, and ABA-1 levels found in stool (R^2^ = 0.7484, P < 0.0001, Fig 2B).

Since *A*. *lumbricoides* morbidity is mainly attributed to M&HI infections (> 5,000 epg), we used this correlation to determine a morbidity cut-off for ABA-1. Based on the linear regression between ABA-1 levels and DNA copies in stool, a morbidity cut-off for ABA-1 of 18.2 ng/g stool was determined, being the minimal ABA-1 concentration that corresponds to M&HI infection. Of the 71 *A*. *lumbricoides* infected individuals studied here, 19 were found to have a M&HI infection based on qPCR. Of those, 13 had ABA-1 levels above this cut-off, corresponding to a sensitivity of 68.4%. Of the 455 samples with no or low intensity infection, only 13 were found to have ABA-1 levels above this cut-off, corresponding to a specificity of 97.1%. Interestingly, 12 false positives had low intensity infection, and only one was negative for *A*. *lumbricoides*, reconfirming the high specificity of the ABA-1 ELISA.

To further establish the role of ABA-1 as a biomarker for *A*. *lumbricoides* infection, stool samples were analyzed from 22 patients with *A*. *lumbricoides* infection who were treated with albendazole. Samples were collected at 0, 6, 12 and 24 days after treatment. qPCR analysis on these stool samples showed complete disappearance of STH DNA in all subjects, indicating killing or expulsion of the parasite (S3 Fig). The quantification of ABA-1 levels in the different stool samples revealed a strong reduction in ABA-1 levels, most of them dropping below LLOQ already at 6 days post treatment. Only 3 out of 22 (13.6%) subjects had ABA-1 levels above the LLOQ at day 12 and 2 out of 22 (9.1%) were positive at day 24 (Fig 2C). These results further establish the value of ABA-1 as a means to monitor *A*. *lumbricoides* infection.

**Fig 3:**
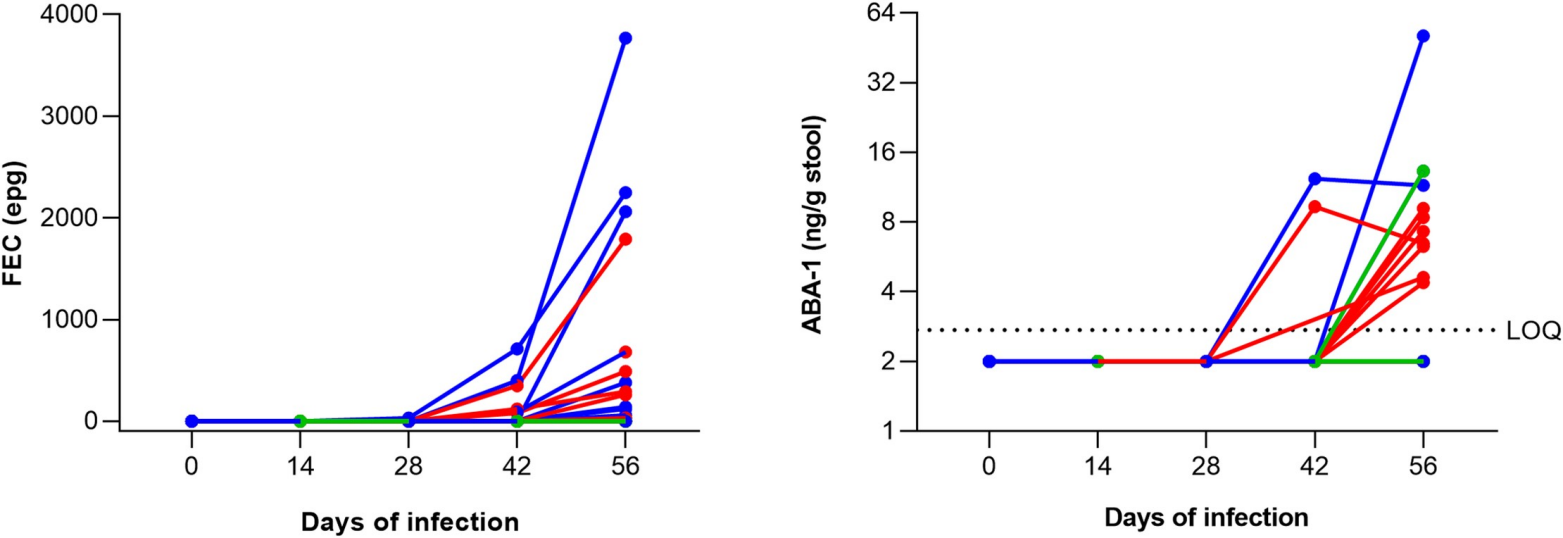
Quantification of fecal egg count (**A**) and ABA-1 in stool (**B**) from *A*. *suum* infected pigs: control group (green), low (red) and high (blue) trickle infected pigs. Stool samples were collected before infection (Day 0) and at different timepoints during trickle infection: 14 days, 28 days, 42 days and 56 days.

Since trickle infection of pigs with *A*. *suum* is a good model to study *A*. *lumbricoides* infection [27], ABA-1 levels were determined in stool samples collected from 25 pigs during the course of the infection: 5 pigs were uninfected (control), 10 pigs were experimentally inoculated with 20 *A*. *suum* eggs/day (low trickle) and 10 pigs were experimentally inoculated with 100 *A*. *suum* eggs/day (high trickle). In all collected stool samples, both fecal egg counts (FECs) and ABA-1 levels were determined (Fig 3). On day 0, 14 and 28 none of the pigs had detectable ABA-1 levels in stool. However, on day 42, two pigs (10%) became positive for ABA-1 and on day 56 this was further increased to 10 pigs (50%). These data might suggest that also in pigs, ABA-1 is a biomarker for *Ascaris* infection. It might however require longer follow-up studies or higher infection intensities to clearly determine the relationship between ABA-1 in stool and *Ascaris* infection in pigs.

## Discussion

The accurate detection of *A*. *lumbricoides* infection is crucial for epidemiologic studies as well as for STH control and elimination programs [5]. Copromicroscopy for detection of *Ascaris* eggs in fecal samples, as well as qPCR-based detection of worm DNA in fecal samples, are the only currently applied techniques to diagnose this gastrointestinal infection [7, 25]. Microscopy lacks accuracy and is operator dependent, while qPCR requires specific laboratory infrastructure and currently lacks standardization [25, 28–30]. Detection of specific serum antibodies to *A*. *suum* has been evaluated using ELISA, but as for any serologic assay that detects the presence of serum antibodies, such a test may not necessarily indicate a current infection [31–33].

The main aim of this study was to develop a sensitive coproantigen ELISA test for *A*. *lumbricoides*, based on the detection of ABA-1 in stool. The ABA-1 ELISA had a LLOQ of 2.74 ng/g stool. Evaluation of this test on a panel of stool samples from school-aged children in Kenya with known parasitological status demonstrated a 95.3% specificity and a 91.5% sensitivity for *A*. *lumbricoides* detection. The assay that was developed not only resulted in a qualitative assessment of infection but was also shown to quantitatively reflect infection intensity. As such, a cut-off of 18.2 ng ABA-1/g stool in this experimental set-up was determined to correspond to the boundary between low and moderate infection intensity. This cut-off is of particular interest as *Ascaris*-related morbidity is predominantly associated to M&HI infections [2]. This is also why the WHO has set the goal to reduce the prevalence of preschool and school-aged children with STH infections of M&HI below 2% by 2030 [3]. Using this cut-off, 68.4% of the subjects with M&HI infection, as determined by qPCR, were correctly identified. Although this might seem suboptimal, it is important to emphasize that in the current study the number of M&HI infection was only 19, with most of them being just above the threshold for moderate infection. Also, it is known that there is significant variability in egg counts between different aliquots from the same stool sample [34]. Hence, there might also be significant variation in ABA-1 levels. Besides this cross-sectional analysis, a longitudinal analysis was performed on samples collected from *A*. *lumbricoides* infected subjects that were treated with albendazole. This treatment typically results in high cure rates, with in most cases no more eggs detected within 7 to 10 days after treatment, something that was observed in the current study as well [35–37]. Similarly, ABA-1 levels also dropped strongly after treatment, with only 13.6% and 9.1% having ABA-1 levels above the LLOQ at 12 days and 24 days post-treatment, respectively. The very low ABA-1 levels observed after 24 days in some subjects may be attributed to worms which survived treatment, but with reduced or cessated egg production (drug-induced embryostasis), a phenomenon already demonstrated for *Onchocerca volvulus* and *A*. *suum* [38, 39]. Alternatively, this could also be caused by some remaining ABA-1 in the gut or possibly even from a new worm infection.

Additional demonstration of the role of ABA-1 as biomarker for *Ascaris* infection was achieved by assessing ABA-1 levels in pigs that were subjected to trickle infection with *A*. *suum*, which is often used as a model to study the host-parasite relationship. Since in the ABA-1 region that was used for the generation of the polyclonal antibodies there is only one amino acid difference between *A*. *suum* and *A*. *lumbricoides* (99.6% identity), it was expected that the antibodies would cross-react with *A*. *suum* ABA-1. It was found that at day 42 of the infection, the first pigs started to shed eggs in their stool and similarly, the first ABA-1 positive stool samples were detected at day 42. Two weeks later, already 50% of the pigs were ABA-1 positive, while 60% were positive by Mini-FLOTAC, indicating that ABA-1 appearance is closely related to the appearance of eggs in stool. This latter observation, together with the observation that bead beating was required to release ABA-1 from stool for ELISA testing, might support the hypothesis that ABA-1 in stool is mainly associated with parasite eggs or egg fragments. The concurrent appearance of ABA-1 and eggs in stool of *A*. *suum* infected pigs is different to what was observed with the *Trichuris vulpis* porin coproantigen assay where coproantigen was detected as early as 23 days post-infection while eggs were not observed before day 69 [9]. It can’t be excluded that the current ABA-1 ELISA lacks sensitivity and that a more sensitive assay might be needed for detection of prepatent infection. In order to further investigate whether ABA-1 is associated to eggs, it could be of interest to study both male and female worm extracts, or to investigate the presence of ABA-1 in stool from individuals that were found to be infected only with male worms (as shown upon expulsion of the worms).

The biggest advantage of a coproantigen test could be its use at the collection site. When school-based deworming programs are being executed, the teams on the ground could use such a test to immediately assess the prevalence and intensity of *A*. *lumbricoides* infection in that community. Integration of school-based surveillance for STH in the deworming programs or other NTD surveillance programs, might be a very cost-effective approach [40, 41]. Target product profiles for such use-case however describe increased reliability in lower transmission settings compared to microscopy [5]. It will require further evaluation to determine whether the coproantigen ELISA described here meets this criterium. The current protocol of the ABA-1 coproantigen test is based on the release of ABA-1 in the fecal samples using bead beating, followed by ELISA to determine the presence and amount of ABA-1 in the extract. Although the bead beating step appears to be essential, it might be of interest to further investigate how this procedure could be simplified and possibly performed in the field. Some simpler technologies to replace the bead beating step are available and could be explored [42, 43]. To release the ABA-1 from stool, we might use strong chaotropic reagents, such as guanidium hydrochloride, which has been shown to be effective in lysing *Mycobacterium tuberculosis* and even plant cell walls [44, 45]. The fact that ABA-1 is a lipid-binding protein might suggest that certain detergents might enable the release of ABA-1 from the fecal samples without the need of bead beating [19]. The second step in the protocol, the ELISA based detection could easily be adapted to a lateral flow immunoassay (LFIA). Similar LFIA’s have been developed for detection of *M*. *tuberculosis* antigens in sputum samples, for the circulating cathodic antigen (CCA) in urine as diagnostic test for *S*. *mansoni*, and many more [46–49]. Also, the antibodies used in the ELISA described here are polyclonal antibodies. The development of monoclonal antibodies might result in an assay with even higher sensitivity and specificity. The current data show that a coproantigen test is a viable option for detection of helminth infection in man. Although we have shown that ABA-1 is a good coproantigen candidate for *A*. *lumbricoides*, there might also be other proteins with an even better sensitivity/specificity profile.

In conclusion, we developed a new sensitive coproantigen ELISA that detects *Ascaris* ABA-1 in stool sample extracts. Using this ELISA, we demonstrated that ABA-1 in stool is a highly sensitive and specific biomarker to detect infection with *A*. *lumbricoides* with ABA-1 levels correlating to infection intensity. A user-friendly and field-adjusted method of this assay could thus potentially be useful in monitoring infection prevalence and intensities in communities during STH control programs.

## Supporting information

S1 Fig Correlation between *A*. *lumbricoides* FECs (expressed in epg) and *A*. *lumbricoides* DNA detection in stool collected in Kenya (expressed in *A*. *lumbricoides* copies/reaction) was used to determine a cut-off of 700 copies/reaction that could be used to identify subjects with moderate-to-high infection. (TIF)

S2 Fig SDS-PAGE (14%) analysis of recombinantly produced and purified His-tagged ABA-1. A total of 2.2 μg of purified protein was loaded and visualized by Coomassie blue staining.
(TIF)

S3 Fig Effect of treatment with albendazole on the presence of *Ascaris lumbricoides* DNA in stool. Stool samples were collected before (Day 0) and at different timepoints after treatment with albendazole: 6 days, 12 days and 24 days after treatment.
(TIF)

S1 Table Performance characteristics of the ABA-1 coproantigen ELISA were determined by analyzing 7 calibration samples (Cal 1–7) with known concentrations of ABA-1 recombinant protein. Calibration curves were analyzed in 4-fold over 3 different days and back-calculated concentrations were used to determine accuracy and coefficients of variation.
(DOCX)

S1 Data BLAST analysis of ABA-1 performed on Wormbase Parasite using the entire *T*. *trichiura* proteome (prjeb535) as database. (TXT)
