# Supplementary figures and images for: Detection of Ascaris lumbricoides infection by ABA-1 coproantigen ELISA
Source: PLoS Negl Trop Dis. 2020 Oct 15;14(10):e0008807. doi: 10.1371/journal.pntd.0008807 (PMC7591086; doi:10.1371/journal.pntd.0008807)

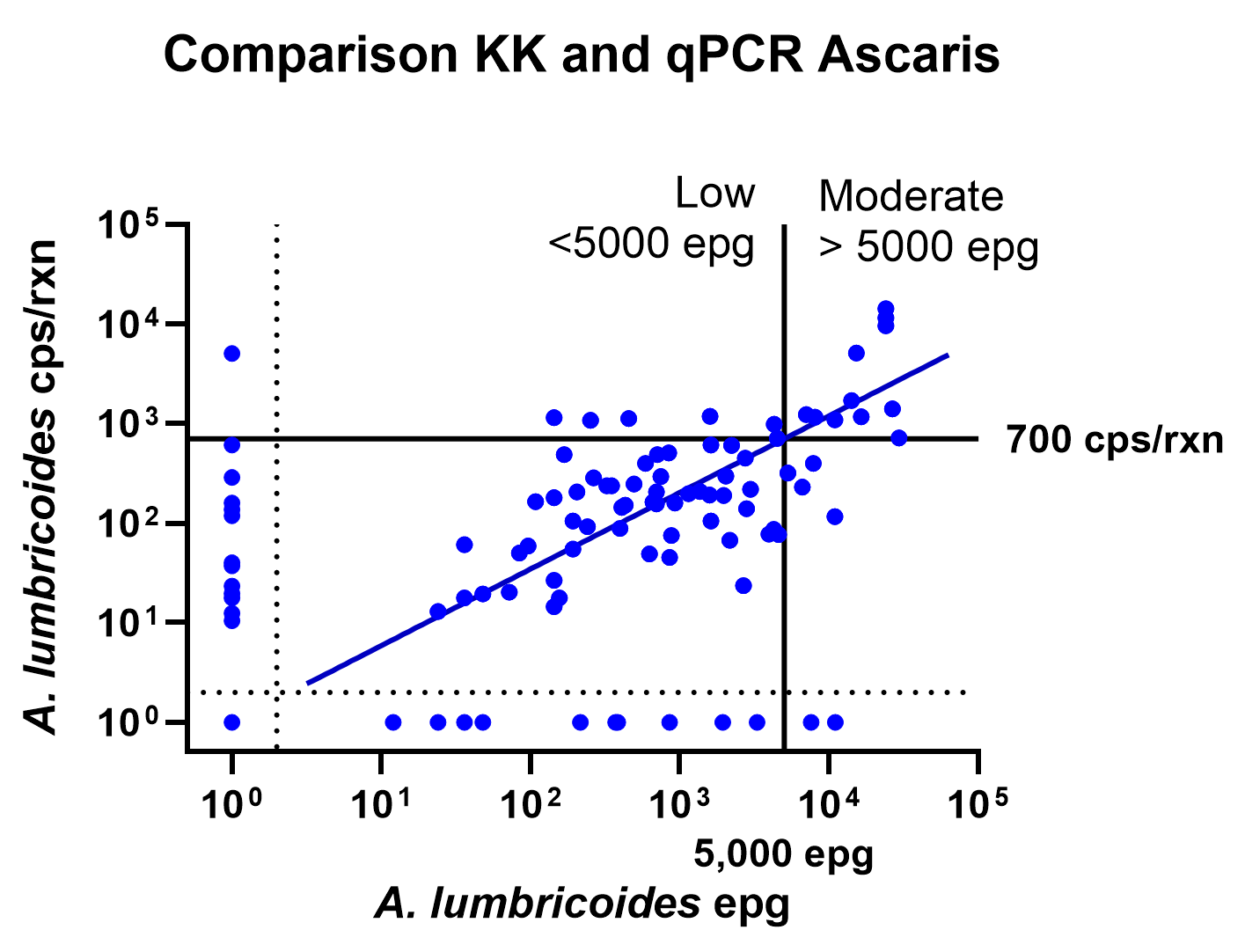

Supplement: S1 Fig — (TIF) [file pntd.0008807.s001.tif]

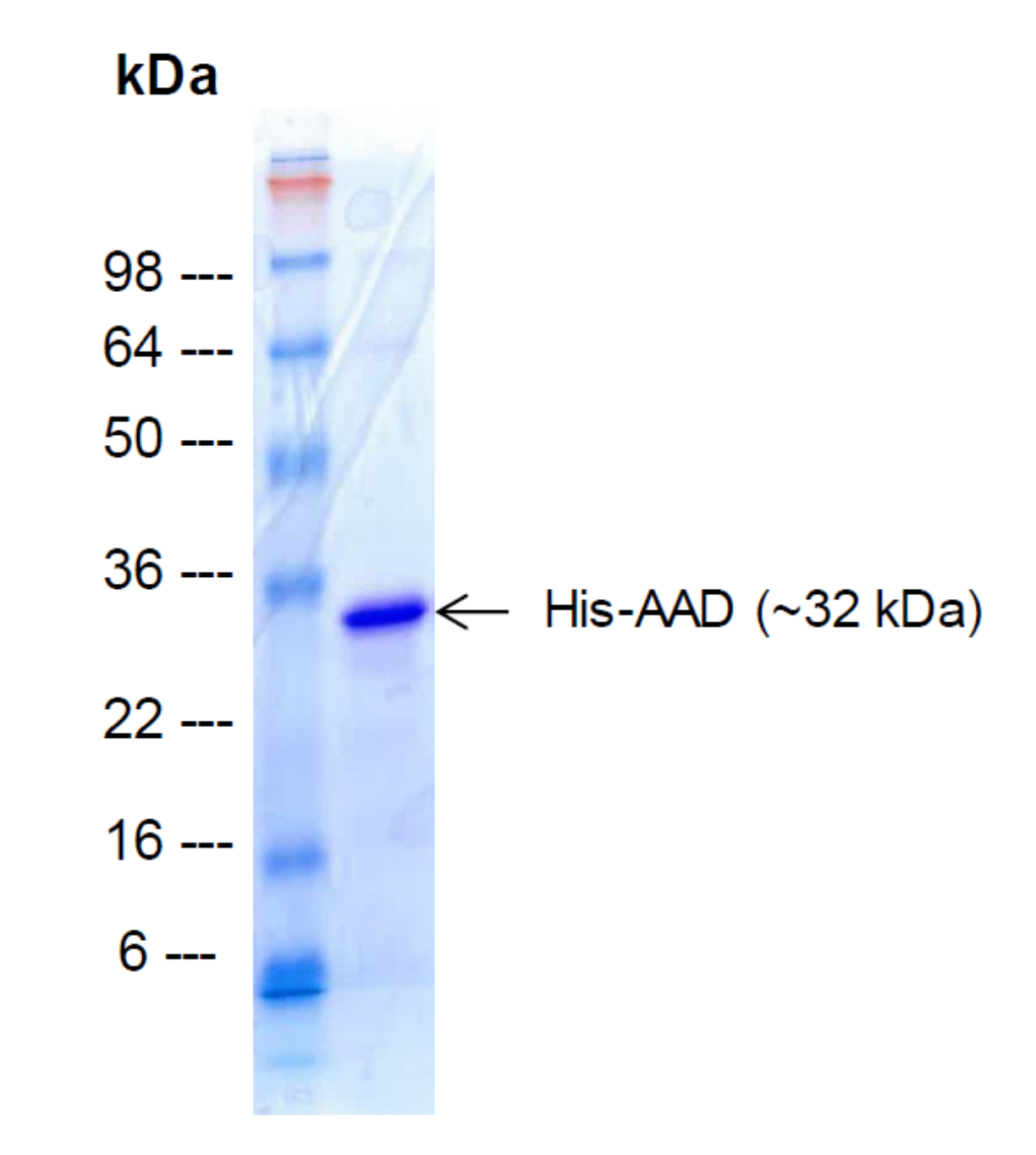

Supplement: S2 Fig — A total of 2.2 μg of purified protein was loaded and visualized by Coomassie blue staining. (TIF) [file pntd.0008807.s002.tif]

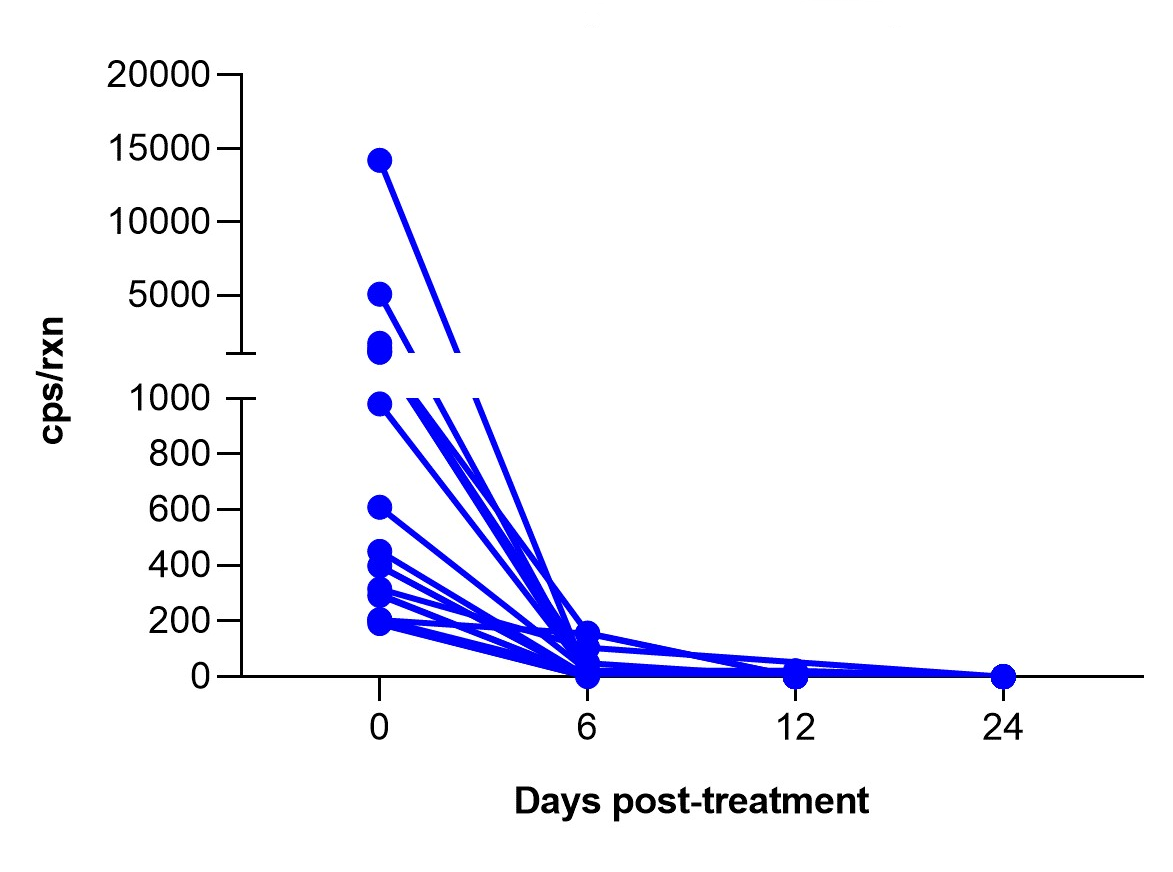

Supplement: S3 Fig — Stool samples were collected before (Day 0) and at different timepoints after treatment with albendazole: 6 days, 12 days and 24 days after treatment. (TIF) [file pntd.0008807.s003.tif]
