# Supplementary material for: Detection of Ascaris lumbricoides infection by ABA-1 coproantigen ELISA
Source: PLoS Negl Trop Dis. 2020 Oct 15;14(10):e0008807. doi: 10.1371/journal.pntd.0008807 (PMC7591086; doi:10.1371/journal.pntd.0008807)
Supplement: S1 Table — Calibration curves were analyzed in 4-fold over 3 different days and back-calculated concentrations were used to determine accuracy and coefficients of variation. (DOCX) [file pntd.0008807.s004.docx]

**S2 Table**. Performance characteristics of the ABA-1 coproantigen ELISA were determined by analyzing 7 calibration samples (Cal 1-7) with known concentrations of ABA-1 recombinant protein. Calibration curves were analyzed in 4-fold over 3 different days and back-calculated concentrations were used to determine accuracy and coefficients of variation.

|  | ABA-1  (ng/mL) | Overall accuracy | Within- run CV | Between-run CV |
| --- | --- | --- | --- | --- |
| Cal7 | 100 | n.d. | n.d. | n.d. |
| Cal6 | 33.3 | n.d. | n.d | n.d. |
| Cal5 | 11.1 | 82.9% | 4.9% | 12.0% |
| Cal4 | 3.70 | 98.4% | 1.0% | 2.0% |
| Cal3 | 1.24 | 96.3% | 0.9% | 2.3% |
| Cal2 | 0.412 | 102.8% | 1.7% | 3.4% |
| Cal1 | 0.137 | 74.1% | 7.8% | 12.9% |

n.d.: not determined as signal is saturated at the 2 highest calibration points.

CV: Coefficient of variability
